# Spectroscopic Evidence of Free Radicals Generated by Indocyanine Green Under Light Irradiation

**DOI:** 10.3390/molecules31183171

**Published:** 2026-09-09

**Authors:** Magdalena Szpunar, Łukasz Dubiel, Bogumił Cieniek, Ireneusz Stefaniuk, David Aebisher, Andrzej Wal

**Affiliations:** 1 Doctoral School at the University of Rzeszów, University of Rzeszów, Al. Tadeusza Rejtana 16C, 35-959 Rzeszów, Poland; 2 Department of Physics and Medical Engineering, Faculty of Mathematics and Applied Physics, Rzeszów University of Technology, Powstańców Warszawy 12, 35-959 Rzeszów, Poland; l.dubiel@prz.edu.pl; 3 Institute of Materials Engineering, Faculty of Exact and Technical Sciences, University of Rzeszów, Pigonia 1, 35-310 Rzeszów, Poland; bcieniek@ur.edu.pl (B.C.); istefaniuk@ur.edu.pl (I.S.); 4 Department of Photomedicine and Physical Chemistry, Faculty of Medicine, University of Rzeszów, Warzywna 1A, 35-310 Rzeszów, Poland; daebisher@ur.edu.pl; 5 Institute of Physics, Faculty of Exact and Technical Sciences, University of Rzeszów, Pigonia 1, 35-310 Rzeszów, Poland; anwal@ur.edu.pl

**Keywords:** indocyanine green, electron paramagnetic resonance, spin trapping, reactive oxygen species, free radicals, DMPO adducts, bovine serum albumin, protein binding, photodynamic therapy, type I photodynamic mechanism

## Abstract

Reactive oxygen species (ROS), particularly free radicals involved in type I photodynamic mechanisms, are key mediators of photodynamic therapy. Indocyanine green (ICG), a clinically approved near-infrared dye, is increasingly being considered as a photosensitizer. It has attracted considerable interest due to its photophysical properties and affinity for serum albumin, which enhances its stability, circulation time, and tumor accumulation. This study aimed to characterize the formation of free radicals generated by ICG upon light irradiation, with particular emphasis on oxygen radicals involved in type I photodynamic mechanisms. Electron paramagnetic resonance (EPR) spectroscopy combined with the spin trap DMPO (5,5-dimethyl-1-pyrroline-N-oxide) was used to identify radicals formed during irradiation with an OSL2 fiber-optic illuminator providing white light. The detected radical species were identified by analysis of their characteristic hyperfine splitting constants and comparison with simulated EPR spectra corresponding to the DMPO-OH, DMPO-OOH, and DMPO-H adducts. Changes in radical concentrations over time were evaluated using the integral intensity of the EPR signals at two ICG concentrations. Differences in EPR signal intensities for samples containing ICG alone and ICG in the presence of bovine serum albumin (BSA) demonstrated the influence of protein binding on radical generation. Additionally, spectral analysis based on a Hamiltonian spin model was applied to support the reliable identification of the observed radical species. These findings provide insight into the free radical pathways of ICG and contribute to a better understanding of its type I photodynamic activity.

## 1. Introduction

Free radicals occur naturally in the body; they are the result of normal cellular metabolism [1]. Radicals are chemical species (atom, molecule or ion) containing one or more unpaired electrons in a valency shell, and they show high reactivity [2]. Reactive oxygen species (ROS) and reactive nitrogen species (RNS) comprise important groups of reactive species involved in numerous physiological and pathological processes [1]. ROS include both free radical and non-radical oxygen-containing species. Among the radical ROSare superoxide radicals ($O_{2}^{•-}$), hydroxyl radicals (•OH), peroxyl radicals (ROO•) and alkoxyl radicals (RO•). Superoxide is the primary reactive oxygen species and precursor of most ROS. Superoxide is unstable and reacts quickly with neighboring molecules and atoms [3]. Superoxide can undergo dismutation, forming hydrogen peroxide (H_2_O_2_), which, although not a free radical, serves as a precursor to the highly reactive hydroxyl radical (•OH) via metal-catalyzed reactions such as Fenton or Haber–Weiss processes [4,5].

The hydroxyl radical is the most reactive ROS and can induce immediate oxidative damage to lipids, proteins, and DNA. Due to its extremely short lifetime, •OH causes site-specific damage near its generation site [6,7].

Free radicals are vital for cellular signaling, immune function, and homeostasis, but their excess leads to oxidative stress, damaging lipids, proteins and DNA. This contributes to diseases such as cancer, diabetes, cardiovascular and neurodegenerative disorders, and aging-related conditions [1]. To manage free radical levels, organisms rely on antioxidant defenses, including enzymes such as superoxide dismutase and catalase, as well as non-enzymatic agents like glutathione, vitamins and uric acid [8].

Photosensitizers (PSs) can be classified into two groups based on the distinct mechanisms by which they generate reactive oxygen species. Type I photosensitizers rely on electron transfer between the excited triplet state (T1) of the PS and surrounding oxygen or substrates, producing free radicals such as superoxide anion ($O_{2}^{•-}$) and hydroxyl radical (•OH). In contrast, type II photosensitizers generate singlet oxygen (^1^O_2_) through energy transfer from the T1 state of the PS to nearby oxygen molecules [9,10].

Compared with type II photosensitizers, which constitute the majority of known PSs, type I photosensitizers reduce their dependence on oxygen by enabling oxygen cycling via superoxide dismutase, making them more effective for treating hypoxic solid tumors. Moreover, the hydroxyl radical (•OH), which possesses the strongest oxidative capability, is considered the principal anticancer agent in type I photodynamic therapy [10,11].

Indocyanine green (ICG) is a nontoxic, near-infrared (NIR) fluorescent dye, approved by the U.S. Food and Drug Administration (FDA), allowing its use in clinical practice. It is used to determine cardiac output, to assess hepatic function and blood flow, and also for ophthalmic angiography [12,13,14]. Following intravenous administration, ICG rapidly binds to plasma proteins, primarily albumin, and is therefore retained in the vascular space. This agent, with a half-life of approximately 150 to 180 s, is eliminated exclusively via hepatic uptake and biliary excretion [14,15].

The non-covalent binding of ICG to human serum albumin (HSA) prolongs its circulation time in the bloodstream. Albumin acts as an endogenous nanocarrier in vivo, stabilizing ICG and increasing its clinical utility. Due to its molecular weight (~67 kDa), albumin readily penetrates leaky tumor vessels and accumulates in the tumor interstitium. HSA limits the aggregation of ICG molecules and attenuates concentration-dependent self-quenching of ICG fluorescence [16]. Consequently, the HAS-ICG complex exhibits a markedly enhanced near-infrared fluorescence signal [17,18]. This complex accumulates in tumors through the enhanced permeability and retention effect, a phenomenon associated with leaky tumor vasculature and impaired lymphatic drainage. In addition, tumors actively uptake albumin as a metabolic substrate, facilitating further intratumoral accumulation [16,19,20].

Spin trap 5,5-dimethyl-1-pyrroline N-oxide (DMPO) is widely used for detecting free radicals through electron paramagnetic resonance (EPR) spectroscopy. This is due to its properties, which include low redox activity and the fact that the spectrum of adducts that are produced after reaction of radicals with DMPO can be distinguished from each other using literature bases [21]. This can be done by analyzing the splitting constants that characterize the shape of the EPR signal.

**Figure 1:**
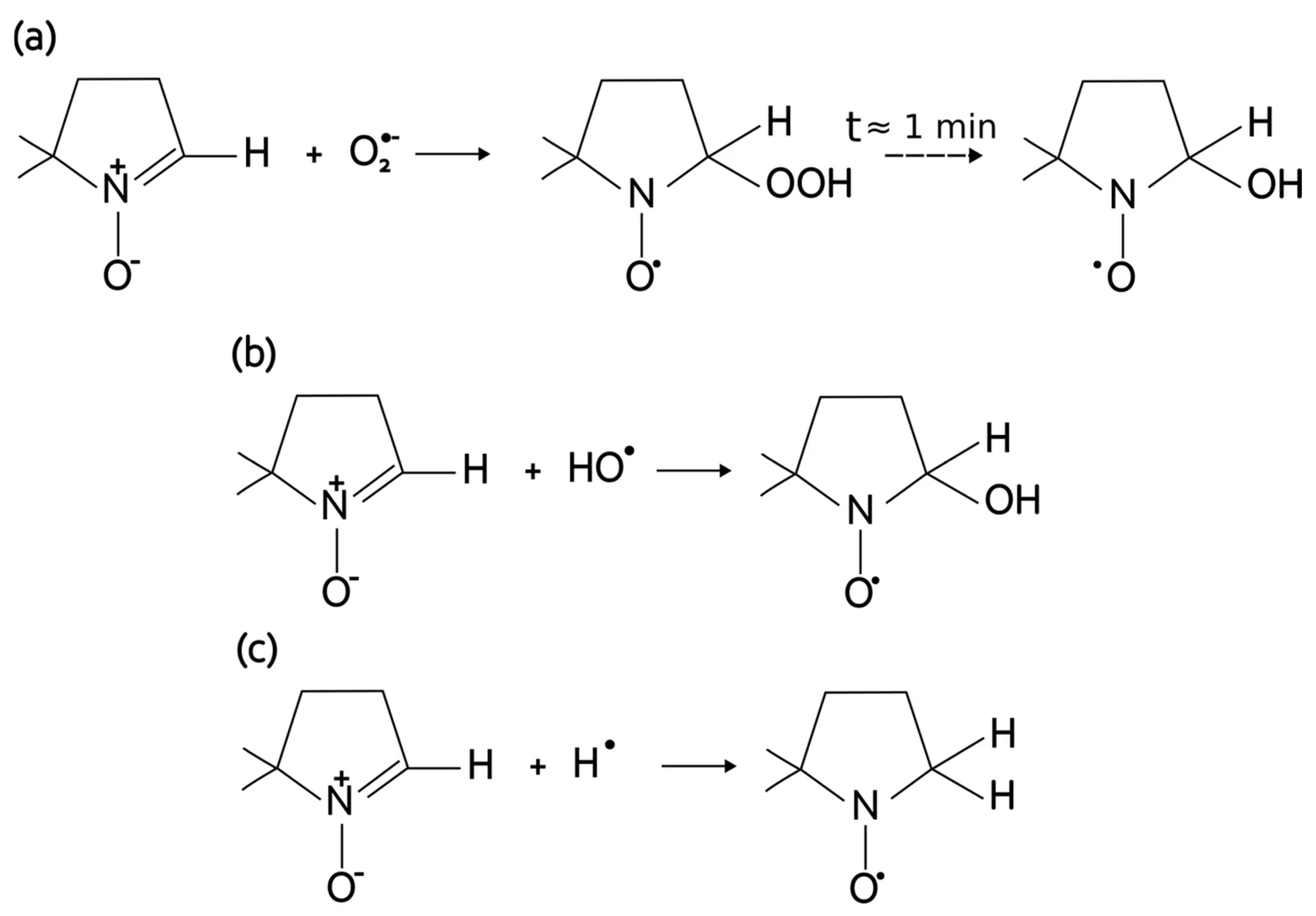
Formation of (**a**) DMPO-OOH, (**b**) DMPO-OH and (**c**) DMPO-H adducts. The dashed arrow in part (**a**) of the figure indicates that the OOH adduct has a lifetime of approximately 1 min, after which it is converted to the OH adduct.

When $O_{2}^{•-}$ and $HO^{•}$ radicals are trapped by DMPO, they form hydroperoxyl DMPO-OOH and hydroxyl DMPO-OH spin adducts, respectively, with different spectral parameters [22]. It is also possible to observe adduct DMPO-H as a result of interaction of $H^{•}$ with the spin trap [23]. A schematic illustration of the spin-trapping method is presented in Figure 1.

The aim of this study was to provide direct experimental evidence of radical species generated by indocyanine green under light irradiation using EPR spin trapping, to identify the nature of the radicals formed, and to investigate the influence of dye concentration and albumin binding on radical formation and their temporal evolution.

## 2. Results and Discussion

### 2.1. UV-Vis Spectral Analysis

**Figure 2:**
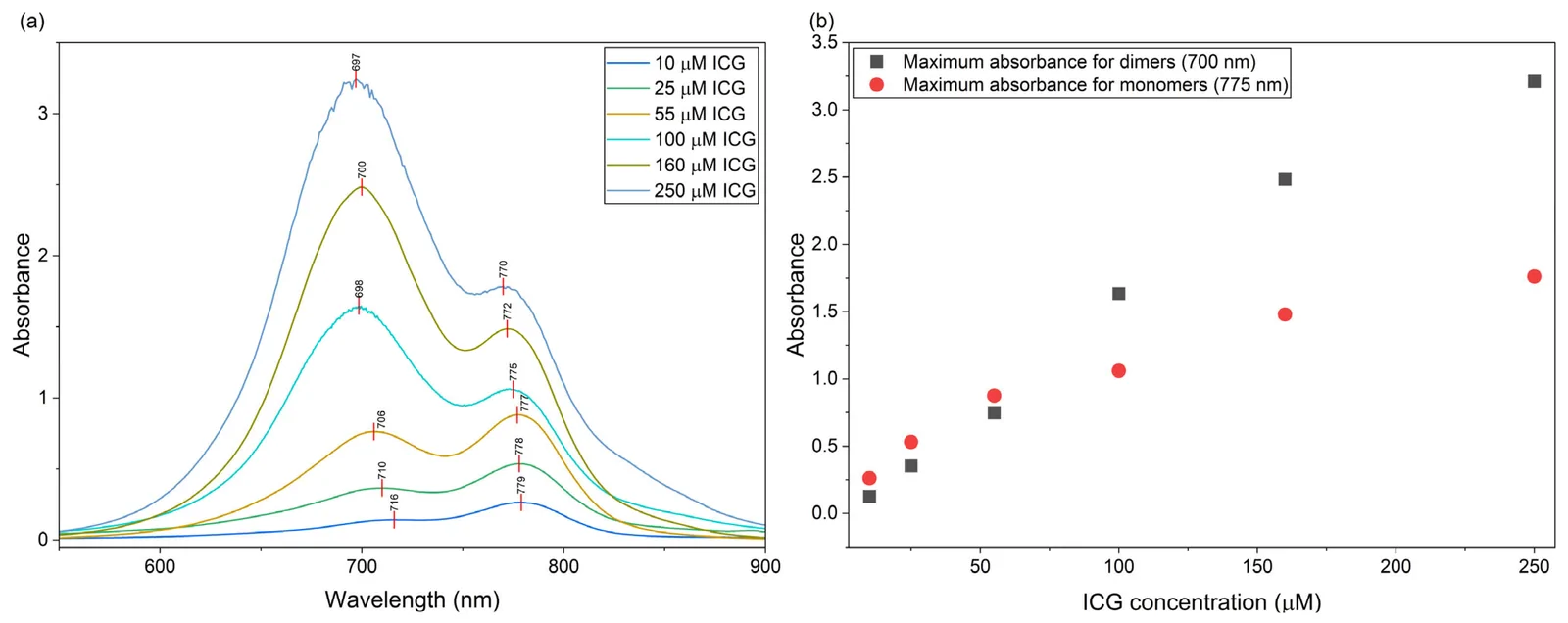
Concentration-dependent changes in the UV-Vis absorption spectra of ICG: (**a**) UV-Vis absorption spectra recorded at ICG concentrations of 10, 25, 55, 100, 160, and 250 μM; (**b**) absorbance measured at 700 and 775 nm as a function of ICG concentration.

To characterize the concentration dependence of ICG aggregation, UV-Vis absorption spectra were recorded over a broad concentration range from 10 to 250 μM (Figure 2a). Increasing ICG concentration resulted in a progressive change in the characteristic absorption bands located in the approximately 700 and 775 nm regions.

For a consistent comparison across the concentration series, absorbance values were extracted at fixed wavelengths of 700 and 775 nm and plotted as a function of ICG concentration (Figure 2b). These wavelengths were selected to approximately represent the characteristic absorption regions associated predominantly with dimeric and monomeric ICG species, respectively. Fixed wavelengths were used because the exact positions of the absorption bands varied slightly with ICG concentration, allowing the spectral changes to be compared consistently across all investigated concentrations.

At 10, 25, and 55 μM, the absorbance at 775 nm was higher than that at 700 nm. With increasing ICG concentration, however, the absorbance at 700 nm increased more rapidly than the absorbance at 775 nm. At 100 μM and above, the absorbance corresponding to 700 nm exceeded the one from 775 nm, with the difference becoming progressively more pronounced at 160 and 250 μM. This concentration-dependent change in the relative intensities of the two characteristic absorption regions is consistent with an increasing contribution of dimeric ICG species at higher dye concentrations.

Based on these spectral characteristics, concentrations of 55 and 160 μM were selected for the subsequent EPR experiments to represent two distinct regimes of ICG aggregation. These concentrations therefore provided an experimentally relevant contrast in the spectral characteristics associated with different aggregation states while maintaining sufficiently strong EPR signals for reliable spin-trapping measurements.

**Figure 3:**
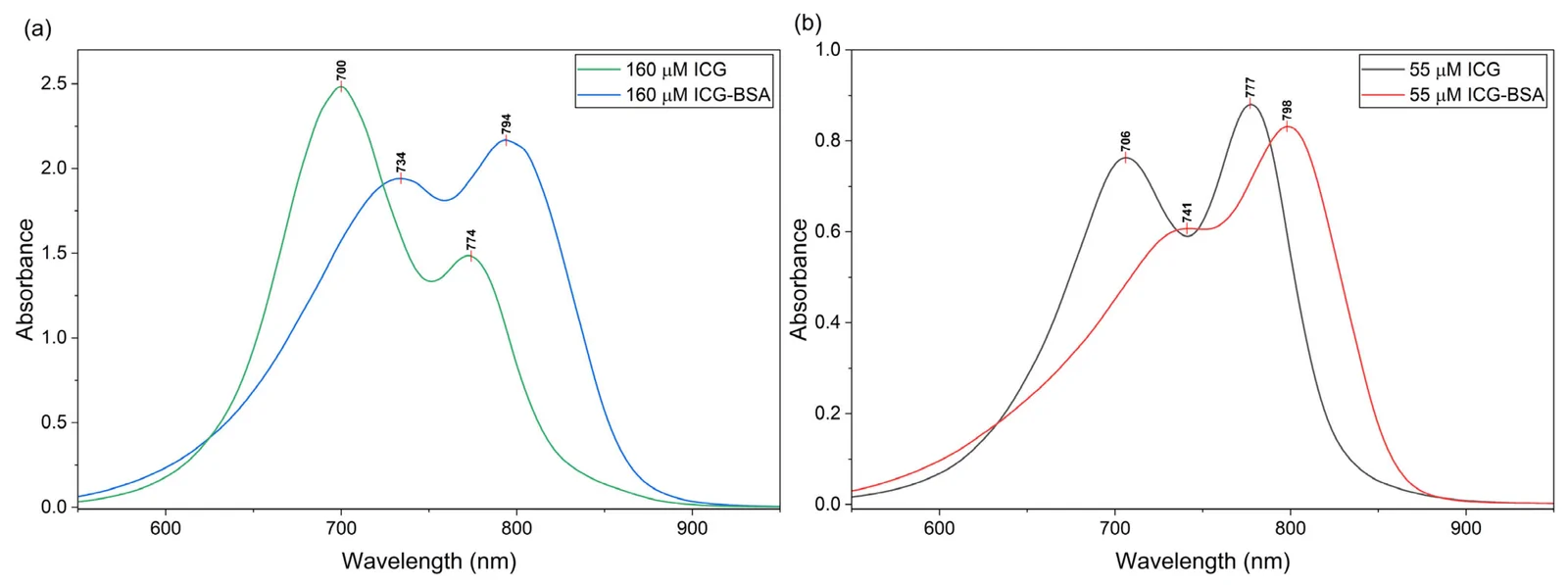
UV-Vis absorption spectra of ICG in aqueous solution and in the presence of BSA at two dye concentrations: (**a**) 160 μM and (**b**) 55 μM. The spectra show characteristic absorption bands corresponding predominantly to monomeric and dimeric forms of ICG.

UV-Vis absorption spectra of ICG and ICG-BSA solutions, presented in Figure 3, were recorded to investigate the influence of dye concentration and bovine serum albumin on the aggregation behavior of ICG.

For 160 μM ICG in aqueous solution (Figure 3a), two main absorption bands were observed at approximately 700 nm and 774 nm, corresponding to dimeric and monomeric forms of ICG, respectively. The presence of both bands indicates the coexistence of aggregated and monomeric species under these conditions. In the presence of BSA, both bands exhibited a red shift to ~734 nm (dimers) and ~794 nm (monomers), accompanied by an increase in the relative intensity of the monomer band. This suggests that albumin binding modifies the dye microenvironment and stabilizes the monomeric form, thereby reducing aggregation [24].

At the lower concentration of 55 μM (Figure 3b), the absorption bands were observed at approximately 706 nm (dimers) and 777 nm (monomers). Upon the addition of BSA, these bands shifted to around 741 nm and 798 nm, respectively. Compared to the 160 μM solution, reduced aggregation was evident at 55 μM, as reflected by the relatively higher contribution of the monomer-associated absorption band.

These results confirm the concentration dependence of ICG aggregation: decreasing dye concentration favors monomer formation, whereas higher concentrations promote dimerization, consistent with previous reports [25]. Overall, both albumin binding and dye concentration significantly influence the aggregation state and spectral properties of ICG in solution.

### 2.2. Spectral Overlap Between OSL2 Light Source Emission and ICG Absorption

**Figure 4:**
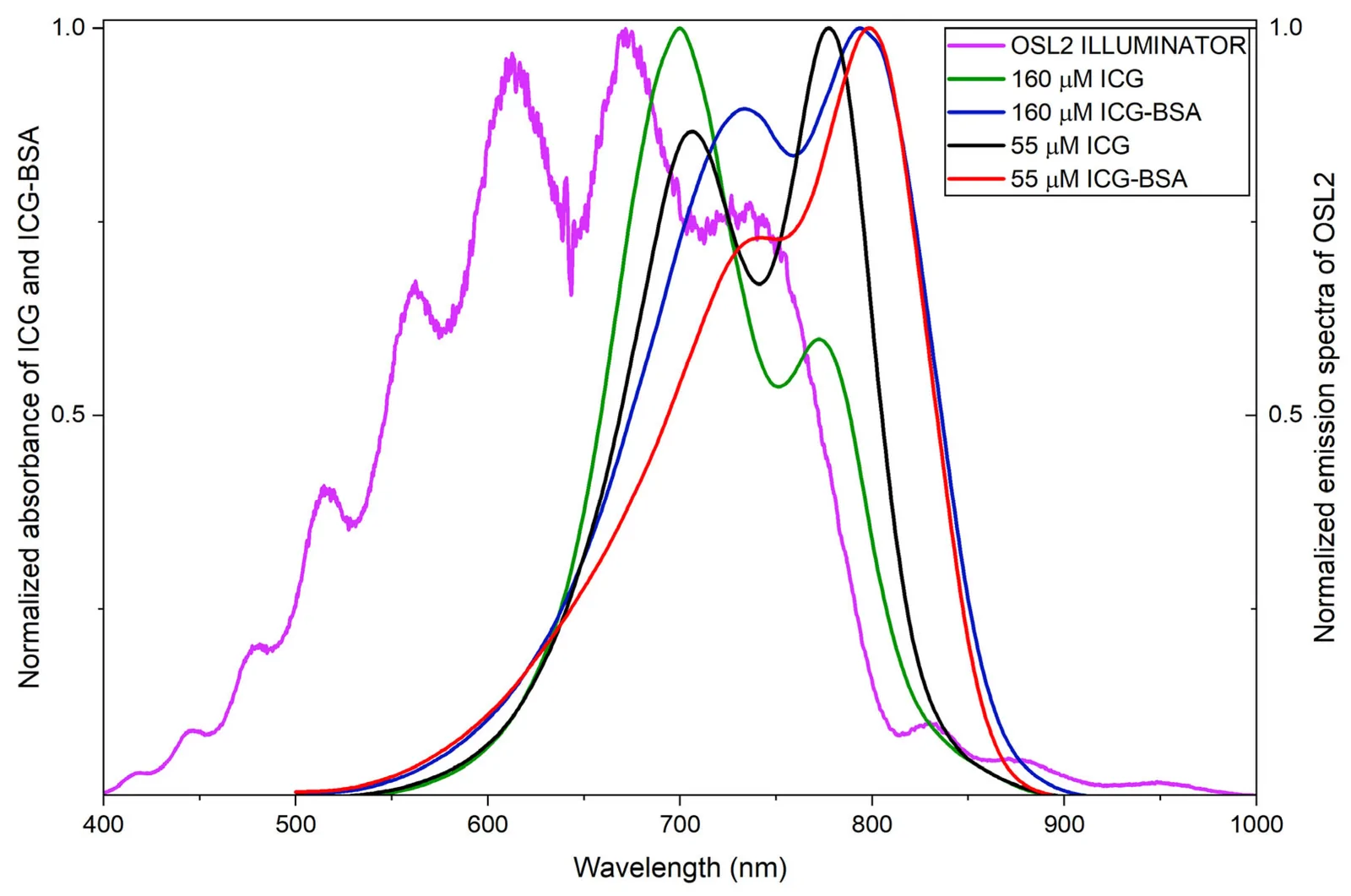
Spectral overlap between the experimentally measured emission spectrum of the OSL2 light source and the UV-Vis absorption spectra of ICG and ICG-BSA.

To further clarify the spectral contribution of the broadband illumination, the experimentally measured emission spectrum of the OSL2 light source was compared with the UV-Vis absorption spectra of ICG and ICG-BSA (Figure 4). The emission spectrum of the OSL2 source substantially overlaps with the characteristic absorption region of ICG, particularly in the approximately 650–800 nm range. This overlap encompasses both the shorter-wavelength absorption band associated predominantly with dimeric ICG species and the longer-wavelength band associated predominantly with monomeric ICG. However, the intensity of the OSL2 emission varies across this spectral range, with a higher emission intensity around the dimeric absorption band and a lower contribution toward the monomeric region.

The spectral overlap indicates that wavelengths corresponding to the absorption bands of ICG are expected to provide the major contribution to direct photoexcitation under broadband illumination. At the same time, because the experiment employed a broadband light source rather than monochromatic irradiation, the present measurements do not allow the photochemical response to be assigned to a single wavelength or the individual contribution of different spectral regions to be quantitatively separated. Wavelengths outside the principal ICG absorption region may contribute to the overall delivered optical energy, but their contribution to direct ICG excitation is expected to be lower where ICG absorption is weak.

### 2.3. Identification of Radical Species by EPR Spin Trapping

In the experiment, radical spin trap DMPO was used, which allowed its selective identification and quantification. Under the influence of light and in the presence of ICG, free radicals are formed and specific adducts are created.

**Figure 5:**
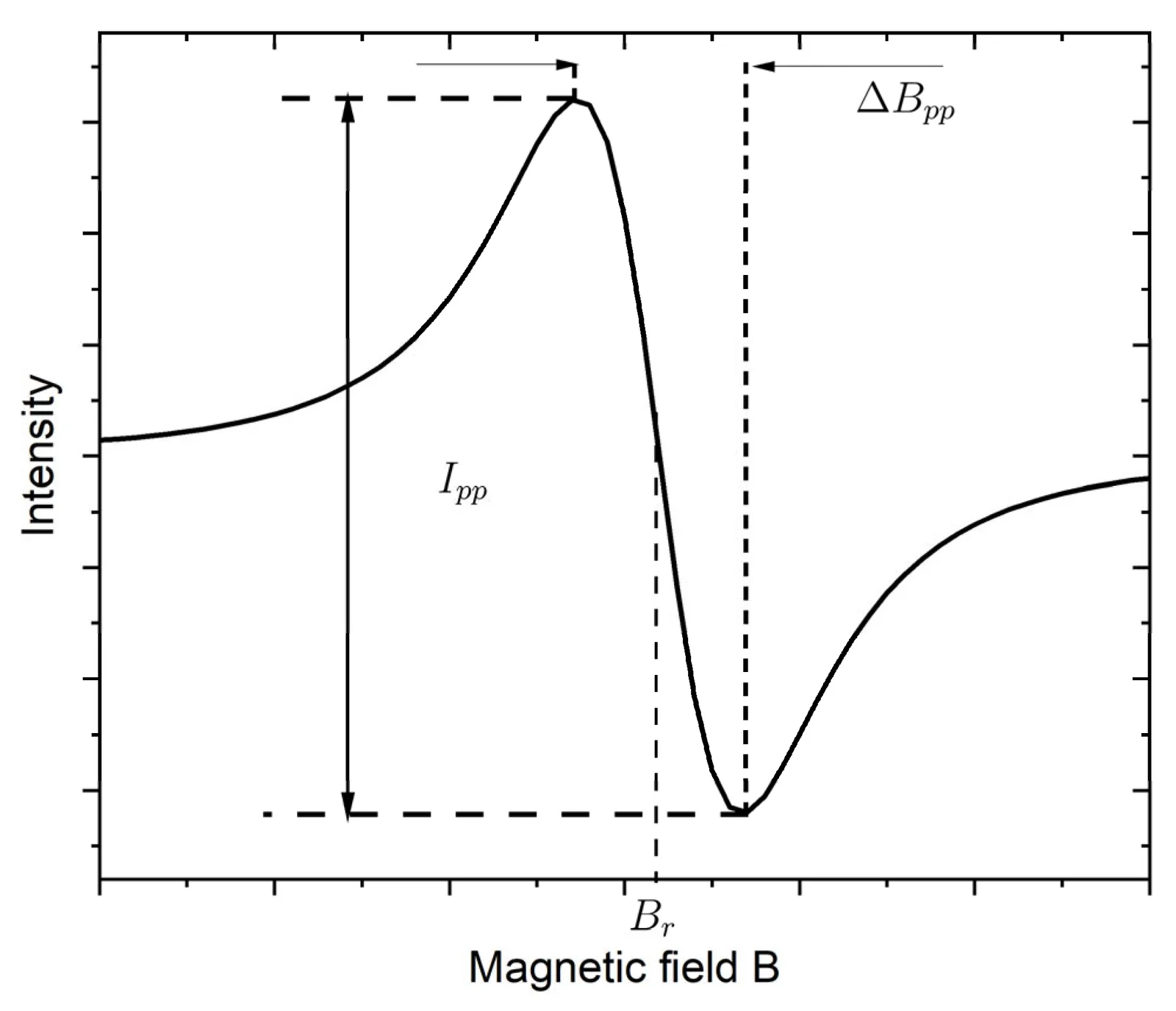
Schematic representation of the derivative of Lorentzian line and its parameters: doubled amplitude of the line $I_{pp}$, peak–peak width $\Delta B_{pp}$ and resonance magnetic field $B_{r}$. Due to the schematic nature of the figure, magnetic field values are not indicated. The dashed guide lines indicate which features of the curve correspond to the individual parameters.

The EPR lines are described by several parameters: doubled amplitude of the line $I_{pp}$, peak–peak width $\Delta B_{pp}$ and the resonance magnetic field $B_{r}$ [26]. The meaning of these parameters for the derivative Lorentzian line shape (typically observed in EPR experiment) is shown in Figure 5.

The important parameter of the EPR line is integral intensity $I_{int}$, which is proportional to the number of spins generating a signal and is calculated as an integral of the absorption line or equivalently as a double integral of the derivative of absorption. It can also be calculated from the following equation [27]:

(1) $$I_{int}=I_{pp}\Delta B_{pp}^{2}.$$

Using this equation, one can calculate the relative (or even absolute number of spins when an appropriate standard is also measured) change in the concentration of radicals generated by indocyanine green upon irradiation.

**Figure 6:**
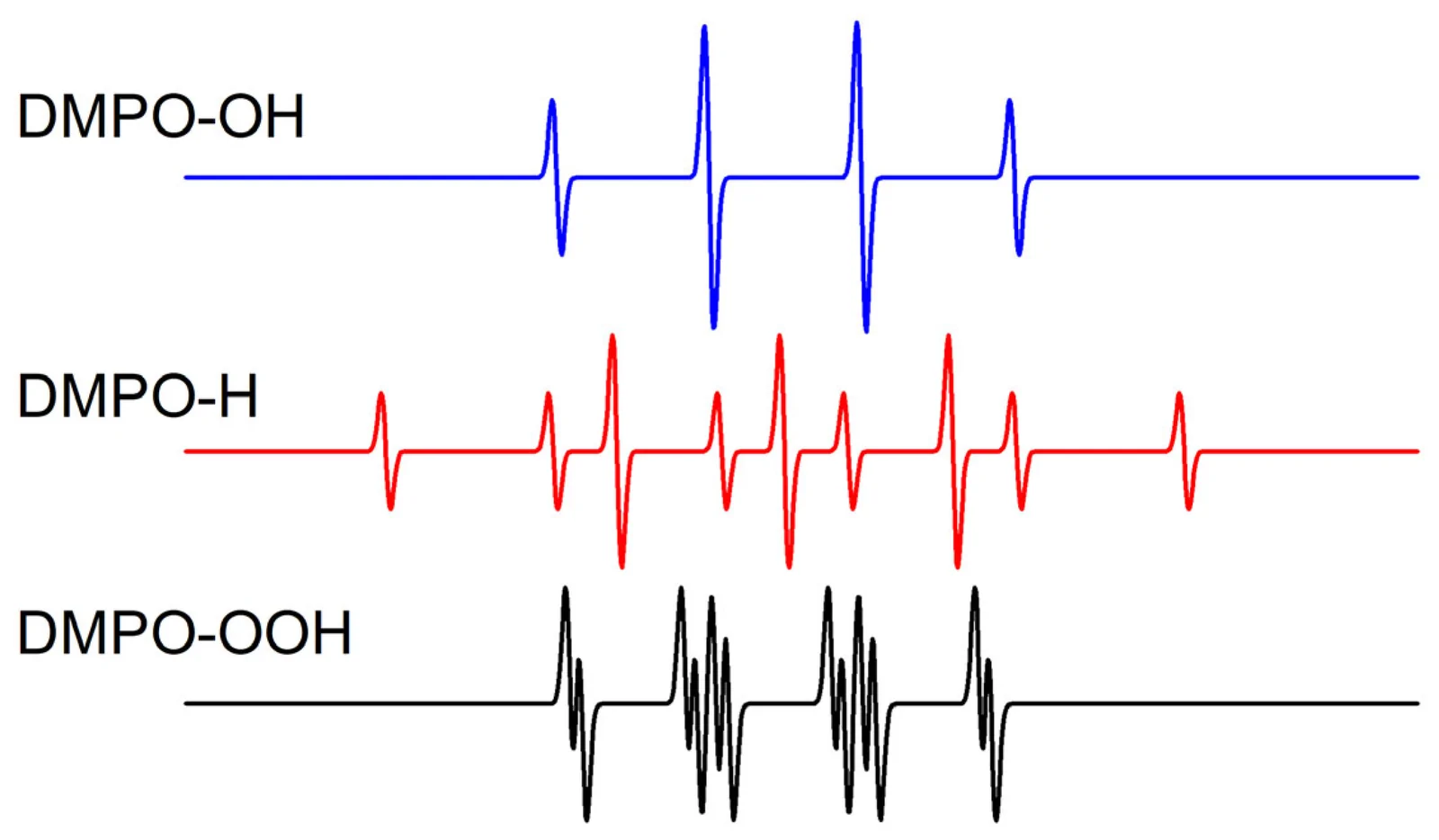
EPR spectral shapes of the individual DMPO adducts. For clarity, the spectra were vertically offset, and the relative signal amplitudes are not shown to scale.

The hyperfine splitting constants for the spectrum of DMPO-OH are $a_{N}=14.9$ G and $a_{H}=14.9$ G, and as a result, the spectrum consists of four lines with relative heights of 1:2:2:1 [22,28]. For DMPO-OOH, the shape of the signal is more complicated than for DMPO-OH, since there are three different hyperfine splitting constants [22,28]: $a_{N}=14.3$ G, $a_{H^{\beta }}=11.7$ G [22] or $a_{H^{\beta }}=11.3$ G [28], and $a_{H^{\gamma }}=1.25$ G. The signal from adduct DMPO-H is characterized by the following constants: $a_{N}=16.6$ G and $a_{H}^{\beta }=22.5$ G [29]. The EPR line shapes of the individual adducts, generated using EasySpin 6.0.12 (Matlab 2023b) software [30] and the parameters specified above, are presented in Figure 6.

**Figure 7:**
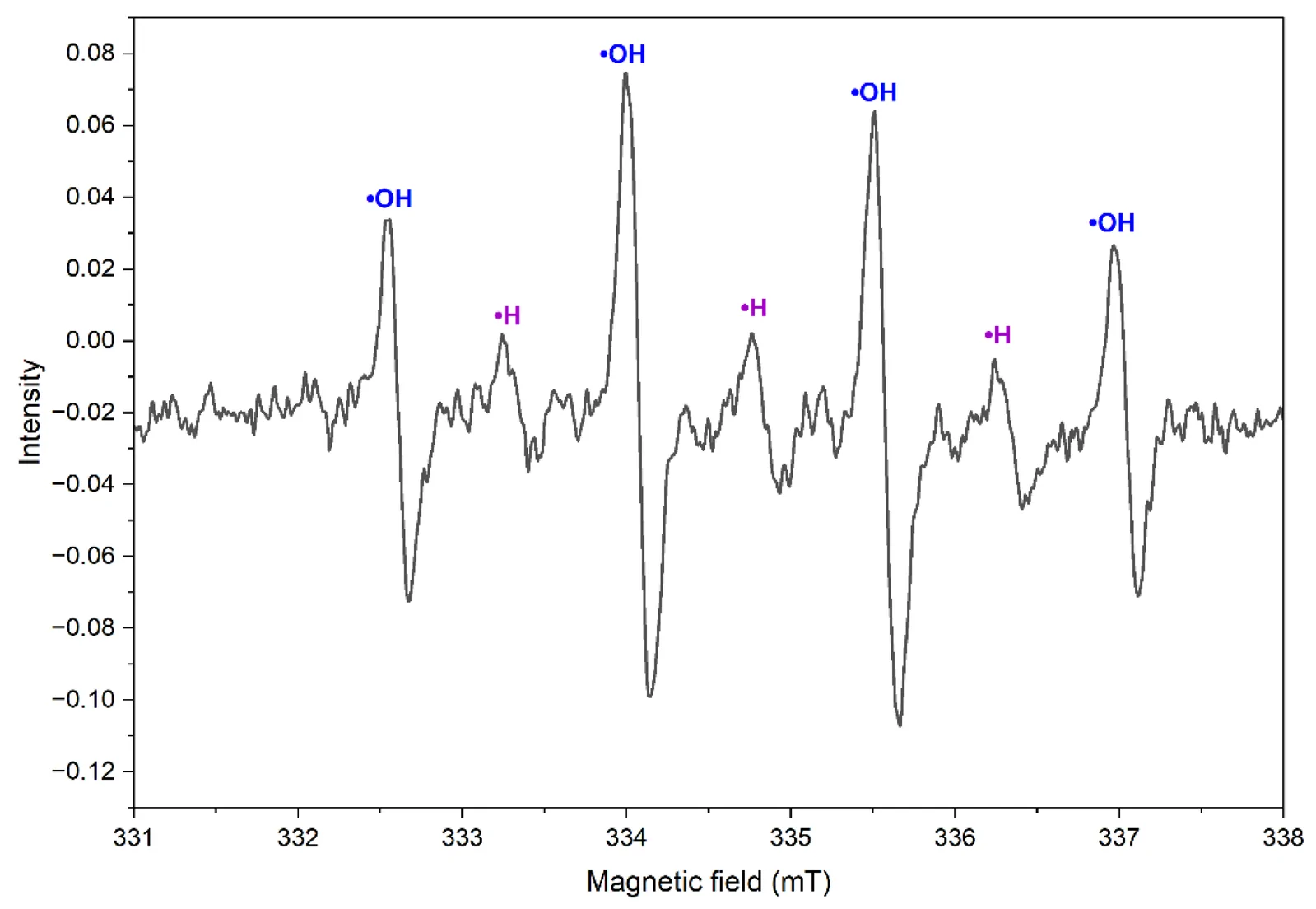
EPR spectrum of 55 μM ICG recorded after 10 min of irradiation in the presence of DMPO. The characteristic signals of the DMPO-OH (•OH, blue) and DMPO-H (•H, purple) spin adducts are indicated.

To evaluate the effects of dye concentration and BSA influence on radical formation, time-dependent changes in the intensities of the EPR signal were analyzed. The focus was placed on DMPO-OH and DMPO-H spin adducts. An example spectrum showing the assignment of spin adducts is shown in Figure 7. The lines marked in blue (•OH, four tallest lines) correspond to the DMPO-OH adduct, while the purple labels (•H) correspond to the three most intense signals originating from DMPO-H.

**Figure 8:**
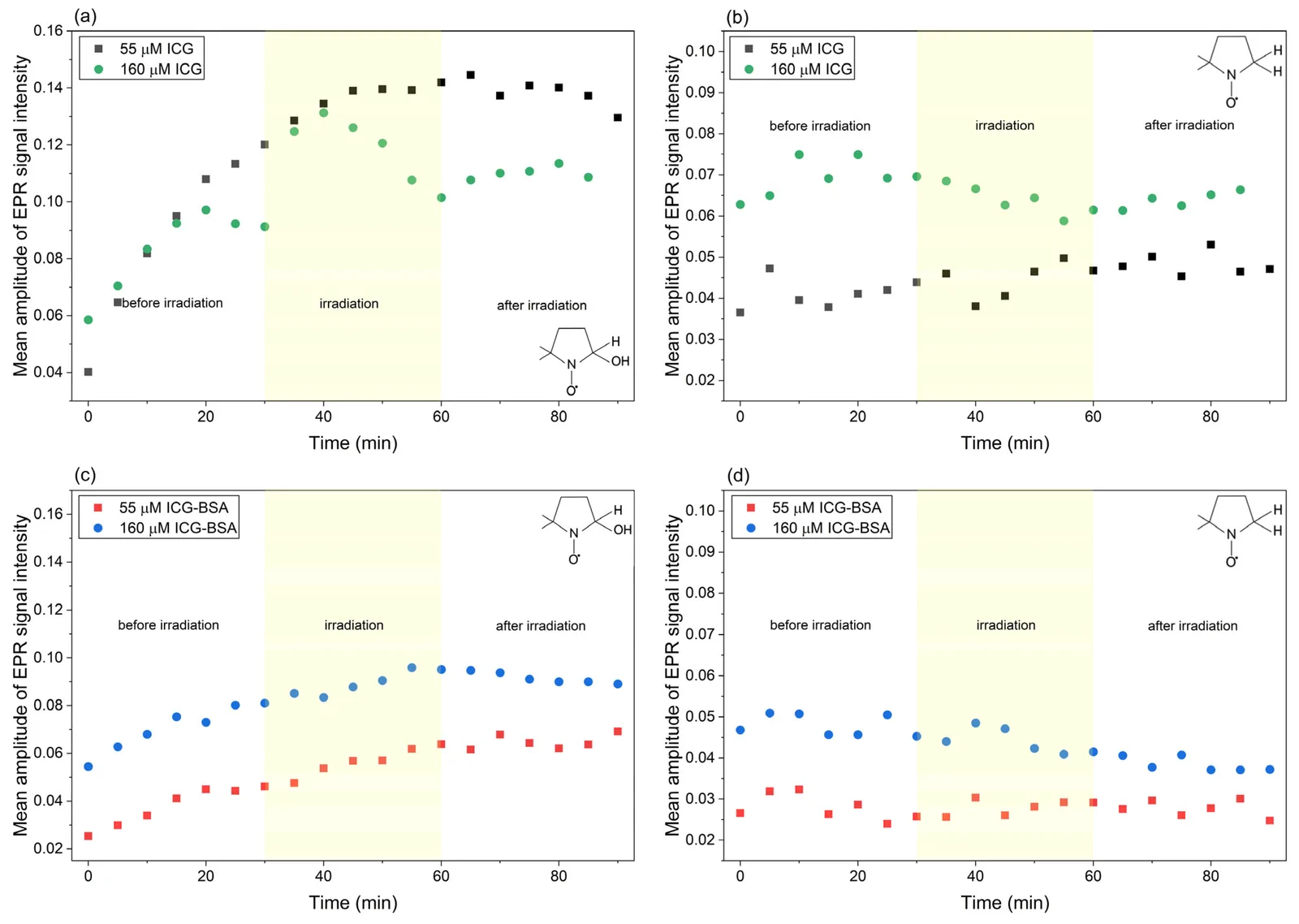
Time-dependent changes in mean EPR signal intensities of DMPO adducts: (**a**) DMPO-OH for 55 μM and 160 μM ICG, (**b**) DMPO-H for 55 μM and 160 μM ICG, (**c**) DMPO-OH for 55 μM and 160 μM ICG-BSA, and (**d**) DMPO-H for 55 μM and 160 μM ICG-BSA.

Time-dependent changes in DMPO adduct intensities are summarized in Figure 8.

For ICG solutions without BSA (Figure 8a), the DMPO-OH signals at both 55 μM and 160 μM increased rapidly during the first 30 min (before irradiation), reaching comparable intensity levels. Upon irradiation, different behaviors were observed depending on concentration. The 55 μM sample continued to exhibit an increase in the intensity of DMPO-OH, although at a reduced rate compared to the initial stage. In contrast, the 160 μM sample showed a decrease in mean EPR signal amplitude of approximately 19% during the 30 min irradiation period. During the post-irradiation stage, both concentrations exhibited relatively stable behavior with a slight decreasing tendency.

The DMPO-H adduct (Figure 8b) showed less regular trends. In the initial period (before irradiation), both concentrations showed a slight increase in signal intensity. Under irradiation, the 160 μM sample exhibited a decreasing tendency, whereas the 55 μM sample showed transient fluctuations, including a short decrease followed by a temporary increase before stabilizing. In the final stage, both samples remained nearly constant with only minor variations.

For ICG-BSA solutions (Figure 8c), the DMPO-OH signal demonstrated a gradual increase throughout the entire experiment for both concentrations, with no clear dependence on irradiation. In contrast, the DMPO-H signal (Figure 8d) showed a slight overall decrease for both concentrations, also without pronounced light-dependent effects.

The observed decrease in DMPO-OH intensity at higher ICG concentration during irradiation may be attributed to enhanced radical recombination processes or increased aggregation of the dye, which can reduce the efficiency of electron transfer pathways. In contrast, continued growth of the DMPO-OH signal at lower concentration suggests more efficient radical generation under reduced-aggregation conditions.

The presence of BSA appears to stabilize the system, leading to more gradual and less light-dependent changes in radical signal intensities. This behavior may result from protein binding, which modifies the local microenvironment of ICG and can limit uncontrolled radical recombination or secondary reactions.

**Figure 9:**
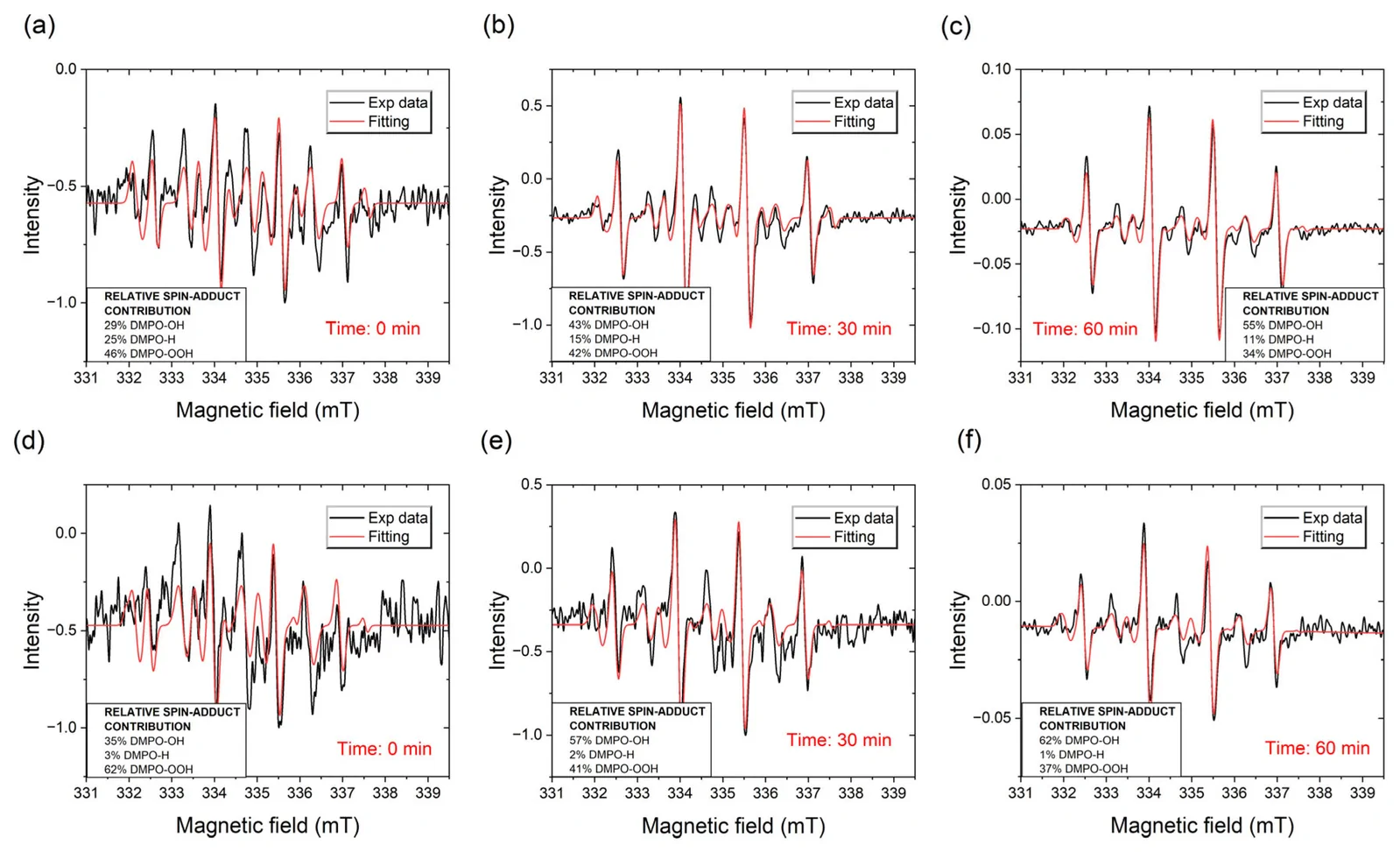
Time evolution of the intensity of EPR spectra recorded during irradiation for ICG (**a**–**c**) and ICG-BSA (**d**–**f**) at concentrations of 55 μM.

The EPR spectra for 55 μM ICG and 55 μM ICG-BSA are shown in Figure 9. All spectra were analyzed and fitted using the EasySpin 6.0.12 (Matlab 2023b) package, based on the spin Hamiltonian formalism [30].

The resulting parameters obtained from the fits, including the g-factors, hyperfine coupling constants ($a_{N}$ and $a_{H}$), and peak-to-peak linewidths $\left(\Delta B_{pp}\right)$ for the DMPO-H, DMPO-OH, and DMPO-OOH adducts, are summarized in Table 1. These parameters reflect subtle changes in the local magnetic and electronic environment of the DMPO spin adducts, likely associated with differences in ICG aggregation and its interaction with the protein matrix. The parameters recorded for DMPO adducts are in agreement with literature data [31].

Initially, i.e., at the beginning of the experiment, the lines corresponding to the different DMPO adducts displayed comparable intensities, with the fitted relative weights of the individual components being approximately 29%, 25%, and 46% for DMPO-OH, DMPO-H, and DMPO-OOH, respectively, in the 55 μM ICG sample. Over time, the signal from the DMPO-OH adduct became dominant, reaching relative weights of approximately 55% and 62% for ICG and ICG-BSA, respectively, at a concentration of 55 μM, indicating enhanced formation of hydroxyl radicals. In addition, a comparison between ICG and ICG-BSA reveals subtle but significant variations in both *g*-factors and hyperfine couplings, particularly for the DMPO-H adduct. The relatively lower contribution of the DMPO-OOH adduct suggests that superoxide-derived intermediates are either short-lived or rapidly converted into hydroxyl radicals under the applied experimental conditions.

**Figure 10:**
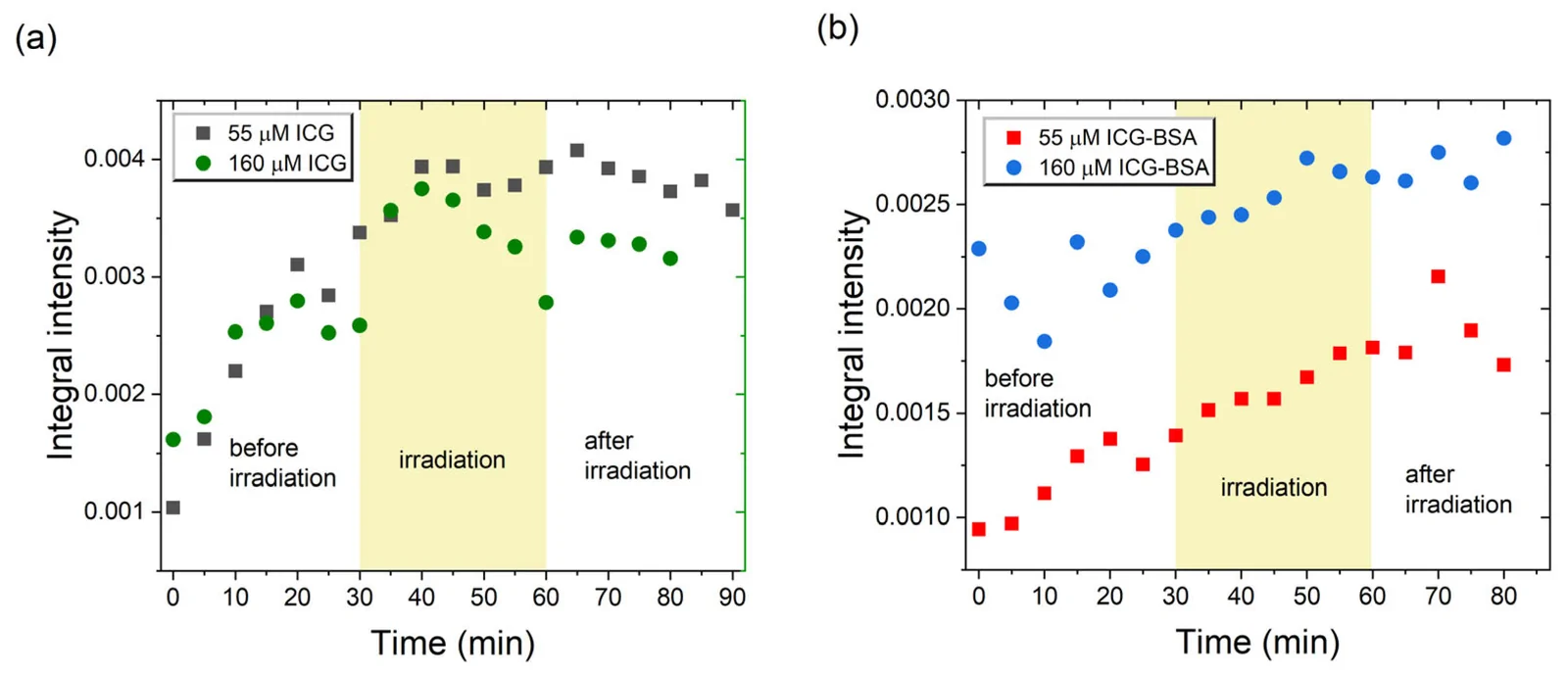
Time-dependent evolution of the integral intensity of the DMPO-OH EPR signal for (**a**) ICG and (**b**) ICG-BSA solutions at concentrations of 55 μM and 160 μM.

The EPR signal parameters obtained from the spectral fitting using EasySpin 6.0.12 (Matlab 2023b) software were used to determine the change in the integral intensity (Equation (1)) during the experiment. The results for hydroxyl radical adducts are presented in Figure 10 and confirm the trends observed in Figure 8.

The time evolution of the integral intensity of the EPR signal of the DMPO-OH adducts reveals clear differences between the systems containing ICG and the ICG-BSA complex. For ICG solutions without BSA, the changes in integral intensity depend on the dye concentration and exhibit different responses to irradiation. For both solutions, a rapid increase in the signal intensity is observed before irradiation. In the case of the ICG-BSA system, the integral intensity values are slightly lower, and the system exhibits a weaker response to irradiation.

Taken together, these observations confirm that the radical species detected by EPR spin trapping are strongly influenced by both dye concentration and the presence of protein. Such effects highlight the importance of molecular aggregation and biomolecular interactions in modulating the photodynamic activity of ICG.

## 3. Materials and Methods

### 3.1. Reagents

Indocyanine green (ICG, United States Pharmacopeia Reference Standard), 5,5-dimethyl-1-pyrroline-N-oxide (DMPO ≥ 98%) and bovine serum albumin (BSA, ≥98%) were purchased from Sigma-Aldrich (St. Louis, MO, USA). Deionized water was obtained from a reverse-osmosis water purification system (Supreme, New York, NY, USA). All reagents and solvents were used as received without further purification.

Stock solutions of ICG (500 μM) and DMPO (250 mM) were prepared in deionized water. BSA was dissolved in deionized water to obtain a stock concentration of 20 mg/mL. Working solutions for UV-Vis and EPR measurements were prepared by mixing appropriate volumes of freshly prepared stock solutions. For samples without BSA, ICG and DMPO were mixed in a 1:2 volume ratio. For samples containing BSA, the volume ratio of ICG:DMPO:BSA was 3:6:2. Final ICG concentrations in the reaction mixtures were 160 μM or 55 μM, corresponding to the four sample compositions. All sample preparations were performed at room temperature.

### 3.2. UV-Vis Measurements

UV-Vis absorption spectra were acquired using a Cary 60 UV-Vis spectrometer (Agilent Technologies, Santa Clara, CA, USA) in the wavelength range of 550–950 nm. Measurements were performed in quartz cuvettes with an optical path length of 2 mm and a volume of 700 μL (Thorlabs, Newton, NJ, USA). Deionized water was used as a blank. All spectra were collected at room temperature.

To characterize the concentration-dependent spectral changes in ICG, an initial series of six ICG solutions in deionized water was prepared at final concentrations of 10, 25, 55, 100, 160, and 250 μM. The obtained absorption spectra were used to evaluate the concentration-dependent changes in the characteristic ICG absorption bands. For comparison across the concentration series, absorbance values at 700 and 775 nm were extracted from the corresponding spectra.

For the subsequent comparison of the effects of ICG concentration and BSA binding, four sample compositions were prepared for UV-Vis analysis, comprising ICG solutions at final concentrations of 160 μM and 55 μM in deionized water, as well as ICG-BSA mixtures at final ICG concentrations of 160 μM and 55 μM, prepared at volume ratios of 3:6:2 and 1:6:2 (ICG:H_2_O:BSA), respectively.

### 3.3. OSL2 Light Source Characterization

The emission spectrum of the OSL2 broadband light source (Thorlabs, Newton, NJ, USA) was measured prior to the experiments using a CCS200/M compact spectrometer (Thorlabs, Newton, NJ, USA) equipped with a CCD detector and dedicated SPLICCO software (version 4.3.73, National Instruments, Austin, TX, USA). Spectral measurements were performed over the wavelength range of 200–1000 nm with a spectral resolution of <2 nm using a 20 μm slit. The measured emission spectrum was subsequently compared with the UV-Vis absorption spectra of ICG and the ICG-BSA complex to assess the spectral overlap between the excitation source and the absorption bands of ICG and to identify the spectral regions potentially contributing to direct photoexcitation.

Optical power was measured with a Thorlabs PM100D power meter coupled to an S425C thermal power sensor (Thorlabs, Newton, NJ, USA). The detector had a circular active surface with a diameter of 2.54 cm, corresponding to an effective area of 5.067 cm^2^.

The OSL2 broadband light source was configured to provide irradiation conditions corresponding to a target fluence of approximately 50 J/cm^2^ over an exposure period of 30 min (1800 s). The light source was positioned at a fixed distance from the detector, and the illuminator output was adjusted accordingly. With the fiber tip positioned 25 cm from the detector and the illuminator operated at 40% output, the optical power measured at the detector surface was 0.141 W.

The irradiance (power density) was obtained by dividing the measured optical power by the sensor’s active area, with this value taken as an approximation of the mean irradiance at the measurement plane:

$$E=\frac{P}{A}$$

where E is irradiance (W/cm^2^), P is the measured power (W), and A is the active detector area (cm^2^). Under the described conditions, the calculated irradiance was 0.0278 W/cm^2^.

The delivered light dose was calculated from the irradiance and exposure duration using

F=E·t

where *F* is fluence (J/cm^2^), *E* is irradiance (W/cm^2^), and *t* is irradiation time (s). For planned irradiation time, the calculated fluence was approximately 50 J/cm^2^ as predetermined.

For an exposure duration of 1800 s, the calculated fluence was 50.04 J/cm^2^, corresponding approximately to the target value of 50 J/cm^2^, which represents a frequently investigated fluence level in PDT studies [32,33,34].

### 3.4. EPR Measurements

Electron paramagnetic resonance measurements were performed in the X-band (ν ≈ 9.4 GHz) using a Bruker ELEXSYS E580 FT-EPR spectrometer (Billerica, MA, USA). The samples were placed in 4 mm outer diameter quartz EPR tubes. Instrumental settings were as follows: sweep width 100 Gs, modulation amplitude 0.5 Gs, and modulation frequency 100 kHz. All measurements were performed at room temperature.

Spin-trapping experiments were performed using 5,5-dimethyl-1-pyrroline-N-oxide (DMPO) as a spin trap. Samples were prepared immediately prior to measurements and placed in the EPR cavity. Each sample was monitored for 30 min prior to irradiation to evaluate signal stability in the dark. Subsequently, samples were irradiated for 30 min and EPR spectra were recorded at 5 min intervals. After irradiation, spectra were recorded for an additional 30 min to monitor post-irradiation changes. The total duration of each experiment was approximately 90 min.

Sample irradiation was performed using a high-intensity fiber-optic illuminator OSL2 (Thorlabs, Newton, NJ, USA) under the irradiation conditions described in Section 3.3. The samples were irradiated for 30 minutes at the fluence of approximately 50 J/cm^2^.

Measurements were performed for four sample compositions: ICG and DMPO mixed in a 1:2 volume ratio at final ICG concentrations of 160 μM and 55 μM, and ICG:DMPO:BSA mixtures at 3:6:2 and 1:6:2, yielding final ICG concentrations of 160 μM and 55 μM, respectively.

## 4. Conclusions

The combined UV-Vis and EPR results demonstrate that the photodynamic behavior of ICG is strongly dependent on the dye concentration and molecular environment. UV-Vis spectroscopy confirmed concentration-dependent aggregation of ICG in aqueous solution, which significantly influences its photochemical reactivity. EPR spin-trapping experiments revealed the formation of DMPO-OH and DMPO-H adducts, confirming the involvement of radical species consistent with a type I photodynamic pathway. The DMPO-OH adduct was found to be the dominant radical signal, indicating that hydroxyl-type radical pathways play a major role under the applied experimental conditions.

At higher dye concentration, enhanced aggregation was associated with altered radical generation kinetics, likely due to modified excited-state properties and increased recombination processes. The presence of BSA further modulated the system, suggesting that protein binding affects both the aggregation state and the formation of reactive oxygen species by creating distinct microenvironments for the sensitizer.

Although the present results clearly confirm the formation of radical species consistent with a type I photodynamic pathway, the relatively moderate light dependence and previously reported singlet oxygen generation [35,36,37] indicate that type I and type II mechanisms may coexist in the ICG system, with their relative contributions depending on the concentration and molecular environment.

These findings highlight that the photodynamic activity of ICG is highly sensitive to its aggregation state and interactions with biomolecular components, which may significantly influence the balance between radical and singlet oxygen pathways. This behavior is particularly relevant for understanding and optimizing the performance of ICG as a photosensitizer in photodynamic therapy applications [38,39,40,41].

## Figures and Tables

**Table 1:** Fitted EPR parameters (*g*-factor, hyperfine coupling constants *a_N_* and *a_H_*, and peak-to-peak linewidth Δ*B_pp_*) of DMPO adducts.

Sample	Adduct	*g*-Factor	*a_N_* (MHZ)	*a_H_* (MHz)	Δ*B_pp_* (mT)
55 μM ICG	DMPO-H	2.006216(25)	44.501(86)	64.138(72)	0.1310(14)
	DMPO-OH	2.006295(13)	41.803(16)	41.297(25)	0.10589(68)
	DMPO-OOH	2.009600(15)	42.338(84)	32.19(11)	0.19883(72)
160 μM ICG	DMPO-H	2.00593(15)	44.921(36)	63.990(39)	0.1476(31)
	DMPO-OH	2.005972(11)	41.772(19)	41.402(25)	0.15129(62)
	DMPO-OOH	2.009333(12)	42.338(84)	32.195(31)	0.1725(16)
55 μM ICG-BSA	DMPO-H	2.006293(25)	46.12(14)	65.138(28)	0.1139(31)
	DMPO-OH	2.00641255(62)	41.726(16)	41.297(15)	0.15357(59)
	DMPO-OOH	2.009677(23)	41.703(52)	32.08(15)	0.2030(13)
160 μM ICG-BSA	DMPO-H	2.00931(13)	45.80(13)	63.73(11)	0.1170(21)
	DMPO-OH	2.005900(15)	41.8222(71)	41.407(20)	0.15188(54)
	DMPO-OOH	2.00931(13)	41.662(47)	33.372(89)	0.2060(11)

## Data Availability

The original contributions presented in this study are included in the article. Further inquiries can be directed to the corresponding author.
